# Phenotype-independent DNA methylation changes in prostate cancer

**DOI:** 10.1038/s41416-018-0236-1

**Published:** 2018-10-15

**Authors:** Davide Pellacani, Alastair P. Droop, Fiona M. Frame, Matthew S. Simms, Vincent M. Mann, Anne T. Collins, Connie J. Eaves, Norman J. Maitland

**Affiliations:** 1 Cancer Research Unit, Department of Biology, University of York, Wentworth Way, York, YO10 5DD UK; 2 Terry Fox Laboratory, BC Cancer Agency, 675 West 10th Avenue, Vancouver, BC V5Z 1L3 Canada; 3 Leeds Institute for Data Analytics, Worsley Building, Clarendon Way, Leeds, LS2 9NL UK; 4 Department of Urology, Castle Hill Hospital (Hull & East Yorkshire Hospitals NHS Trust), Cottingham, HU16 5JQ UK; 5 Hull York Medical School, University of York, Heslington, York, YO10 5DD UK

**Keywords:** Prostate cancer, DNA methylation, DNA methylation, Prostate cancer

## Abstract

**Background:**

Human prostate cancers display numerous DNA methylation changes compared to normal tissue samples. However, definitive identification of features related to the cells’ malignant status has been compromised by the predominance of cells with luminal features in prostate cancers.

**Methods:**

We generated genome-wide DNA methylation profiles of cell subpopulations with basal or luminal features isolated from matched prostate cancer and normal tissue samples.

**Results:**

Many frequent DNA methylation changes previously attributed to prostate cancers are here identified as differences between luminal and basal cells in both normal and cancer samples. We also identified changes unique to each of the two cancer subpopulations. Those specific to cancer luminal cells were associated with regulation of metabolic processes, cell proliferation and epithelial development. Within the prostate cancer TCGA dataset, these changes were able to distinguish not only cancers from normal samples, but also organ-confined cancers from those with extraprostatic extensions. Using changes present in both basal and luminal cancer cells, we derived a new 17-CpG prostate cancer signature with high predictive power in the TCGA dataset.

**Conclusions:**

This study demonstrates the importance of comparing phenotypically matched prostate cell populations from normal and cancer tissues to unmask biologically and clinically relevant DNA methylation changes.

## Background

Treatment-naïve prostate cancer (PCa) is characterised by an abnormal accumulation of proliferative cells with a molecular phenotype similar to the luminal cells present in the normal prostate.^1,2^ However, PCa samples also contain a small population of tumour cells with basal features. These cells possess “cancer stem cell” features, appear to be treatment-resistant, and are proposed to serve as a reservoir for tumour recurrence after castration therapy.^3–6^ DNA methylation of bulk PCa samples has been well studied^7^ and aberrant methylation of promoter regions found to be a consistent feature,^8^ albeit with high variability both between patients and within single tumours.^9^ Their frequency and presence in pre-malignant tissues support a strong selective pressure for DNA methylation changes during cancer development.^7^ However, DNA methylation is dynamically regulated during tissue development and cell differentiation,^10^ and distinct cell types possess specific DNA methylation profiles within the same tissue.^11–13^ Therefore, the luminal molecular features of bulk PCa samples, in contrast to the almost equal proportion of basal and luminal cells in normal prostate tissues, complicate the interpretation of datasets generated on whole tissue extracts, where changes associated to differences in cell types may mask the presence of malignancy-associated signatures.

Recent developments in tissue processing and the identification of surface markers suitable for the prospective isolation of viable basal and luminal cells from normal prostate tissues have enabled studies of their molecular and biological characteristics.^14–17^ Use of this approach has revealed that many of the genes downregulated in normal luminal cells compared to basal cells are frequently hypermethylated in PCa.^18^ These data imply a functional link between DNA hypermethylation and the observed expansion of cells with a luminal phenotype in PCa. However, very little is known about the specific DNA methylation features of PCa cells with basal and luminal phenotypes in comparison to their normal counterparts. To address this issue, we generated genome-wide DNA methylation profiles of FACS-purified populations of cells with basal and luminal features from a series of freshly isolated patient-matched tumour and normal samples from individuals undergoing radical prostatectomy. Our results show that many DNA methylation changes frequently seen in PCa are characteristic differences between luminal and basal cells from both normal and cancer samples. From these datasets, we were also able to identify two sets of tumour-specific changes of potential clinical interest. One set consists of changes that are specific to PCa luminal cells; the other set are changes shared by both basal and luminal tumour but not normal prostate cells.

## Methods

### Tissue processing

Prostate tissues were obtained from patients undergoing radical prostatectomy at Castle Hill Hospital (Cottingham, UK) with informed patient consent and approval from the NRES Committee Yorkshire & The Humber (LREC Number 07/H1304/121). Tissues were sampled immediately after surgery. For radical prostatectomies, three core needle biopsies were taken from four different sites (left base, left apex, right base, right apex) and were directed by previous pathology, imaging and palpation. Tissues were transported in RPMI-1640 with 5% FCS and 100 U/ml antibiotic/antimitotic solution at 4 °C, and processed immediately upon arrival. PCa diagnosis was confirmed by histological examination of the whole prostate. Tissues were disaggregated as previously described,^19^ and all reagents were supplemented with 10 nM R1881 to better preserve the viability of luminal cells.

### Fluorescence activated cell sorting (FACS) and characterisation of cell populations

Single-cell suspensions were labelled with Lineage Cell Depletion Kit (human) and CD31 MicroBead Kit (Miltenyi Biotec) and Lin^+^/CD31^+^ cells depleted twice using MACS LS Columns (Miltenyi Biotec). Lin^−^/CD31^−^ cells were then labelled with EpCAM-APC, CD49f^−^FITC and CD24-PE (Miltenyi Biotec) and DAPI and EpCAM^+^/CD49f^+^/CD24^-^ and EpCAM^+^/CD49f^-^/CD24^+^ sorted at >95% purity using a MoFlo (Beckman Coulter) cell sorter. Sorted populations were characterised by immunofluorescence and qRT-PCR as previously described.^18^

### Reduced representation bisulphite sequencing (RRBS)

DNA was extracted from FACS-sorted populations using phenol/chloroform extraction and ethanol precipitation. DNA was quantified using a NanoDrop 1000 Spectrophotometer (Thermo Fisher Scientific), and shipped to Zymo Research for RRBS analysis. Bisulphite conversion, library preparation, sequencing, and initial bioinformatics analyses were performed by Zymo Research following the Methyl-MiniSeq pipeline.

### Sequence data processing and methylation calls

Fastq files were trimmed using Trim Galore! v0.4.1 (http://www.bioinformatics.babraham.ac.uk/projects/trim_galore/) with the following parameters: --fastqc --illumina --paired --rrbs --non-directional. Trimmed sequences were aligned to the human genome (hg19 downloaded from UCSC, 08-Mar-2009 version) using bsmap v2.90^20^ and the following parameters: -m 0 -x 1000 -n 1 -p 8 -S 1. The resulting bam files were sorted and indexed using samtools v0.1.19,^21^ and methylation and coverage calls for each CpG site calculated using the methratio.py script in the bsmap software (Supplementary Table 1). Methylation calls were then filtered for low (<3) and high (>99.95%) read coverage and merged in non-overlapping genomic bins of 100 bp using the methylKit package v0.99.2^21^ within R v3.3.1 to increase comparability between samples. All subsequent analyses were carried out using only those genomic bins covered in all samples, with the exception of the results presented in Supplementary Fig. 2 and Supplementary Table 3 which were generated using single GpG information.

### Identification of differentially methylated regions (DMRs)

DMRs were calculated using methylKit;^22^ with all pairwise comparisons between the four cell populations carried out and similar populations from different donors defined as biological replicates. The patient of origin was used as a categorical covariate to account for the strong inter-donor variability seen. All *p*-values were generated using a logistic regression model and corrected for multiple testing using the SLIM method.^23^ DMRs were defined as those genomic bins with *q*-values < 0.05 and absolute methylation difference >10% in each pairwise comparison.

### Characterisation of DMRs

All genomic features were downloaded from the UCSC Table browser (genome.ucsc.edu) for the hg19 genome. Gene models: “refGene” (RefSeq Genes), CpG Islands: “cpgIslandExt”, Evolutionary conservation: “phastCons100way”, DNase hypersensitivity sites (DHSs): “wgEncodeRegDnaseClusteredV3”, transcription factor binding sites (TFBSs): “wgEncodeRegTfbsClusteredV3”, repeats: “rmsk” (RepeatMasker). Overlaps and distances of DMRs to other genomic features were calculated using BEDtools v2.26.0^24^, and significance of enrichments or depletions was calculated using custom R scripts. All *p*-values <10^−300^ were approximated to 10^−300^ to avoid reaching the minimum value for a floating-point number (2.2 × 10^−308^). Average conservation signals around DMRs were calculated using bwtool v1.0^25^. *P*-values were calculated using a bootstrapping approach comparing the average conservation of the distal DMRs with the average of an equal number of randomly selected, non-overlapping, distal genomic bins, 1000 times. Gene ontology (GO) analysis was performed using GREAT v3.0^26^, using all covered genomic bins as background and the default “Basal plus extension” association rules. Results were filtered to include only GO categories with a Benjamini–Hochberg corrected (FDR) hypergeometric test *p*-value <0.05 and ≥3 genes with associated regions. K-means clustering of GO categories (biological processes only) was based on information similarity values calculated using the GOSim package within R v3.3.1. Promoters frequently altered in PCa were downloaded from the review by Massie et al.^7^ Only promoters reported by ≥3 studies were considered frequently altered. Genome browser plots were generated using the package Sushi within R v3.3.1 and custom scripts.

### TCGA data analysis

Illumina Infinium HumanMethylation450 data generated within the The Cancer Genome Atlas (TCGA) consortium^27^ were downloaded (pre-processed Level 3 data only) from the NCI Genomic Data Commons website using the provided GDC Data Transfer Tool (data downloaded on 7th Dec 2016). Clinical data were downloaded from firebrowse.org (8th Dec 2016). The presence of evident batch effects was excluded by visualising the data on TCGA Batch Effects (http://bioinformatics.mdanderson.org/tcgambatch/). A data matrix containing the beta values for each sample was generated using custom scripts. Probes were mapped to hg19 using the positions officially reported by Illumina. Overlap of array probes with DMRs was carried out using BEDtools v2.26.0. Hierarchical clustering was based on Euclidean distances of unscaled beta values. Logistic model training using least absolute shrinkage and selection operator (LASSO) regression was performed using the glmnet package within R v3.3.1 on a random selection of 70% of the samples. 200 lambda values ranging from e^−7^ to e^−2^ were tested and 10-fold cross-validation performed. The lambda with the minimum mean cross-validated error was selected and resulted in 17 probes with non-zero coefficients. The optimal model was then tested on the remaining 30% of samples and receiver operating characteristic curve and area under the curve (AUC) calculated using the ROCR package.

## Results

### Phenotypically defined prostate cells from patient-matched normal and PCa samples show donor-specific DNA methylation profiles

**Fig. 1:**
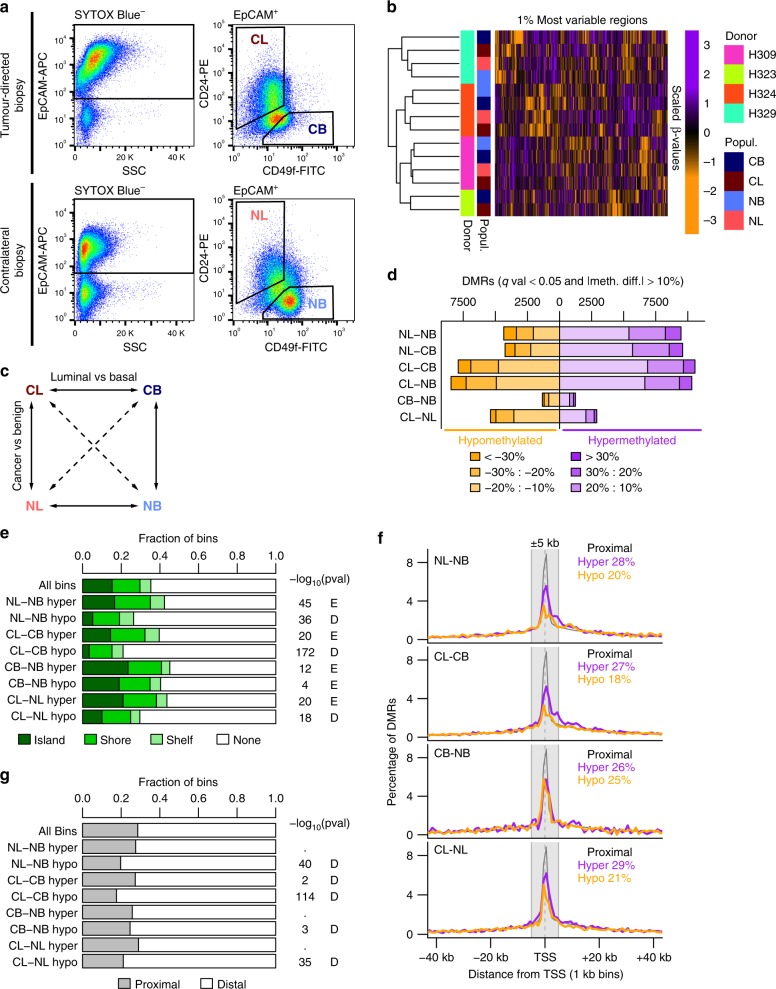
Identification of DMRs between prostate cancer cell populations. **a** Representative FACS profiles of a cell suspension prepared from core needle biopsies of a radical prostatectomy sample. **b** Heatmap showing scaled methylation values of the top 1% most variable regions (100 bp bins) in the samples analysed. Hierarchical clustering is based on Euclidean distance of the unscaled values and complete linkage. **c** Diagram showing all pairwise comparisons carried out. **d** Number of DMRs found in each comparison. **e** Overlap of DMRs with CpG islands, shores (2 kb flanking islands) or shelves (2 kb flanking shores). *P*-values from hypergeometric test against all regions. E = enriched, D = depleted. **f** Distribution of distances of DMRs to the closest TSS. Grey box indicates ±5 kb from a TSS. Purple lines: hypermethylated DMRs, orange lines: hypomethylated DMRs, grey line: all regions. **g** Proportion of DMRs proximal or distal to TSSs. *P*-values from hypergeometric test against all regions. E = enriched, D = depleted

Matched tumour-directed (cancer) and contralateral (normal) core needle biopsies (1 or 2 per site) were obtained from four treatment-naïve prostate cancer patients undergoing radical prostatectomies. These samples were then enzymatically dissociated and labelled with antibodies against EpCAM, CD49f and CD24 to enable the prospective isolation of luminal (EpCAM^+^/CD49f^−^/CD24^+^) and basal (EpCAM^+^/CD49f^+^/CD24^−^) cells at >95% purity (Fig. 1a). EpCAM^+^/CD49f^+^/CD24^−^ cells expressed higher levels of molecular markers associated with basal cells and lower levels of luminal markers compared to EpCAM^+^/CD49f^−^/CD24^+^ cells from the same biopsy, both at the mRNA and protein level (Supplementary Fig. 1A, B). For convenience, we named the paired subsets as follows: Cancer Luminal (CL) EpCAM^+^/CD49f^−^/CD24^+^ cells purified from tumour-directed biopsies; Cancer Basal (CB) EpCAM^+^/CD49f^+^/CD24^−^ cells purified from tumour-directed biopsies; Normal Luminal (NL) EpCAM^+^/CD49f^−^/CD24^+^ cells from contralateral biopsies; Normal Basal (NB) EpCAM^+^/CD49f^+^/CD24^−^ cells purified from contralateral biopsies. This yielded four CL and CB populations, and three matched NL and NB populations, as in one prostate the palpable tumour was extended to most of the prostate and it was not possible to obtain a contralateral “normal” tissue biopsy (Supplementary Fig. 1C). DNA obtained from each of these isolates was then subjected to RRBS. On average, this generated information on the DNA methylation status of >8.9 × 10^6^ cytosines within CpG sites per sample (range 8 × 10^6^–9.6 × 10^6^, with an average coverage of 7.5 reads, Supplementary Table 1). The data were processed as described in Methods, and binned into 100 bp genomic regions to maximise the comparability between samples (932,905 bins covering 4.1 × 10^6^ CpGs in all samples). Unsupervised hierarchical clustering of the top 1% most variable regions (bins) across all samples showed clustering primarily according to the patient of origin, rather than the subset analysed (Fig. 1b). This indicates a high donor-determined variation in CpG methylation, consistent with previous reports of similarly accrued data.^28^

### Distinct DNA methylation profiles in basal and luminal cells

We then calculated DMRs for all pairwise comparisons between the four sorted populations (Fig. 1c, Supplementary Table 2). Among these, the comparison between CB and NB cells (CB-NB comparison) produced the smallest number of DMRs. In contrast, a large number of DMRs were seen when either normal or cancer luminal cells were compared with either source of basal cells (i.e., NL-NB, NL-CB, CL-NB and CL-CB, Fig. 1d). Of the DMRs revealed in these latter comparisons, ~2/3 were hypermethylated in luminal cells, which correlates with the higher levels of DNMT3a seen in these cells.^18^ We also calculated differential methylation on single CpGs (prior the 100 bp binning) with very similar results (Supplementary Fig. 2 and Supplementary Table 3). Moreover, integration of the DMRs identified in NL-NB proximal (±5 kb) to annotated transcriptional start sites (TSSs) with RNA-seq data of similarly purified cells^15^ showed the expected inverse correlation (Supplementary Fig. 3A).

We also found an extensive overlap in the DMRs obtained from both the NL-NB and NL-CB comparisons, and also from the CL-NB and CL-CB comparisons (Supplementary Fig. 3B, C). Accordingly, we focussed our subsequent analyses on comparisons of NL-NB and CL-CB, where cells from the same biopsy could be compared directly.

Characterisation of the genomic features of the DMRs thus identified showed that >50% of them fell outside of CpG islands, shores or shelves (Fig. 1e), and >70% were >5 kb away from any annotated TSSs (Fig. 1f, g). These features were particularly pronounced (highly significant hypergeometric test) for the hypomethylated DMRs identified in the comparisons of NL-NB, CL-CB and CL-NL. Because hypermethylated and hypomethylated DMRs might be anticipated to differ in their genomic context, their impact on the biological properties of basal and luminal cells could also be different.

### Distal hypermethylated DMRs are enriched in enhancer features

**Fig. 2:**
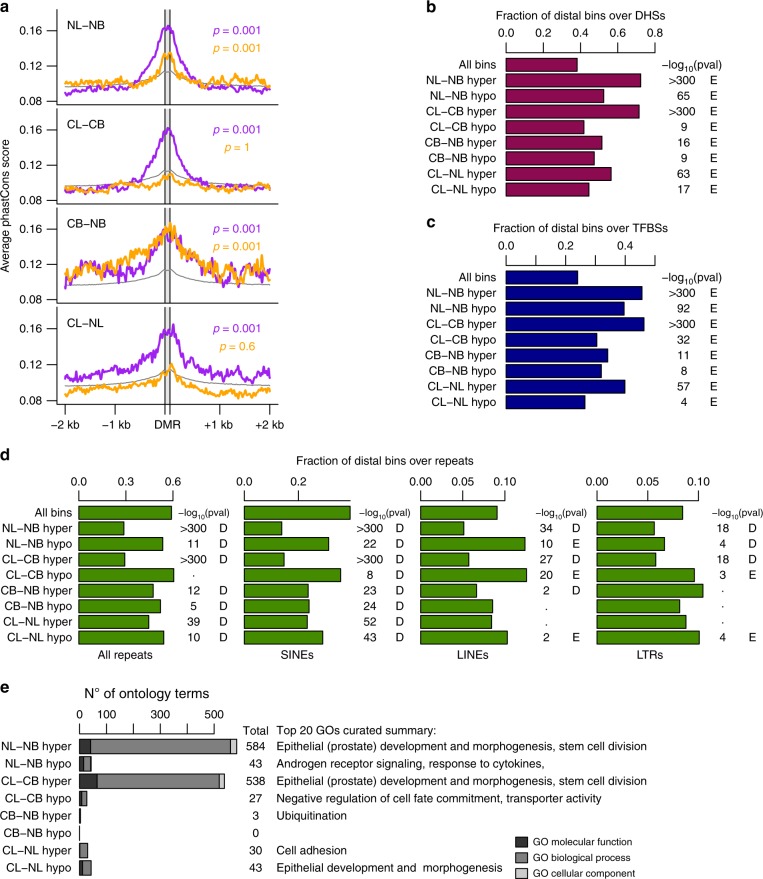
Hypermethylated distal DMRs have features of enhancers. **a** Average plots of evolutionary conservation scores of the distal DMRs in each set. Purple lines: hypermethylated DMRs; orange lines: hypomethylated DMRs, grey line: all regions. *P*-values are from bootstrapping analysis. **b** Proportion of distal DMRs overlapping with DHSs (identified by ENCODE). *P*-values from hypergeometric test against all regions. E = enriched, D = depleted. **c** Overlap of distal DMRs with ChIP-seq derived TFBSs (identified by ENCODE). *P*-values are from hypergeometric tests against all regions. E = enriched, D = depleted. (**d**) Overlap of each set of distal DMRs with repetitive elements (UCSC repeatMask), SINEs, LINEs and LTRs. *P*-values from hypergeometric tests against all regions. E = enriched, D = depleted. **e** Number of GO terms enriched by each set of DMRs. GO terms identified using GREAT (FDR < 0.05 and at least three genes in the set)

Given that most of the DMRs identified were outside CpG islands and far from TSSs, we asked whether they might affect distal regulatory elements (enhancers). We therefore examined three genomic characteristics of such elements: evolutionary conservation,^29^ open chromatin shown by hypersensitivity to DNase I^30^, and presence of TFBSs.^31^ Distal hypermethylated DMRs in each comparison were enriched for evolutionarily conserved sequences (Fig. 2a, bootstrapped *p*-value) and overlapped significantly with both DHSs and ChIP-seq-defined TFBSs (identified within the ENCODE project, Fig. 2b, c, hypergeometric test). Distal hypomethylated DMRs generally scored lower than the hypermethylated counterparts for each metric measured. DMRs hypomethylated in the CL-CB and CL-NL comparisons showed the weakest enrichments. However, all distal hypomethylated DMRs had high overlaps with genomic repetitive elements (Fig. 2d). Specifically, LINE and LTR elements, but not SINE elements, were significantly enriched in the distal CL hypomethylated regions.

GO enrichment analysis (Fig. 2e, Supplementary Fig. 4) showed that hypermethylated DMRs in NL-NB were enriched for more than 500 terms, many of which were linked to prostate development or epithelial stem cell regulation; while hypomethylated DMRs in the same comparison were enriched for terms related to androgen receptor signalling and response to cytokines. In the CL-CB comparison, hypermethylated DMRs were also enriched for more than 500 terms, 311 of which were also identified in the NL-NB comparison, suggesting a high functional overlap in hypermethylated regions in luminal cells from both normal and cancer samples. In the CL-NL comparison, hypermethylated DMRs were enriched in terms related to cell adhesion, while hypomethylated DMRs were enriched in terms related to epithelial morphogenesis. These results indicate that several pathways fundamental to the establishment and maintenance of the normal prostate epithelium are altered in cancer cells with a luminal phenotype.

### Phenotype-specific DMRs are shared in normal and cancerous prostate tissues

**Fig. 3:**
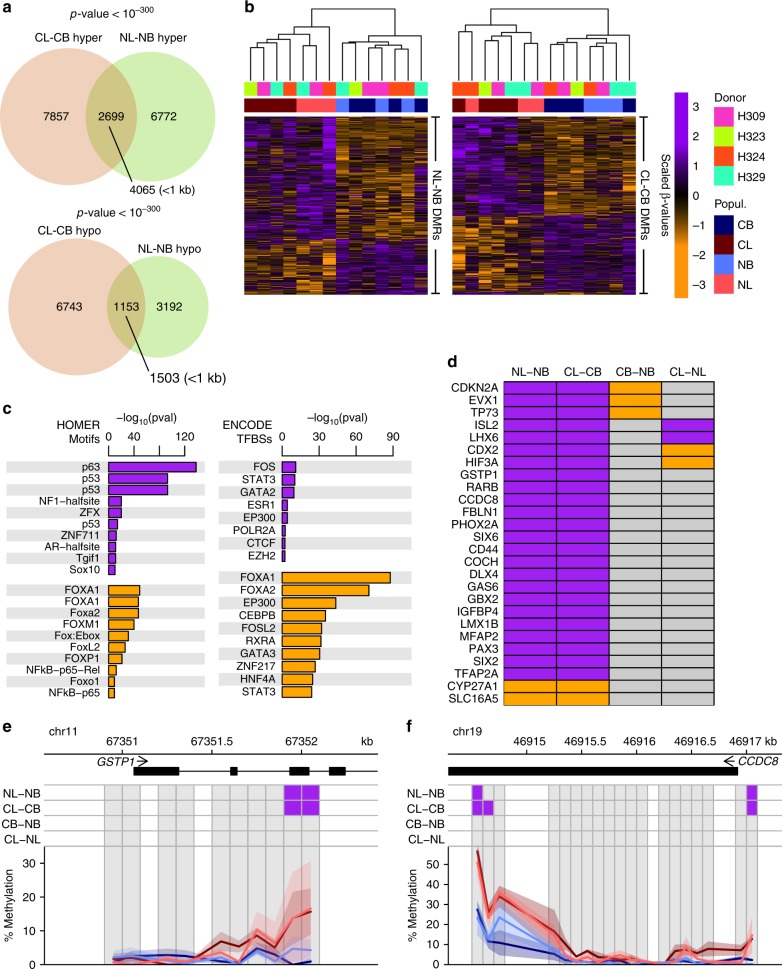
Shared phenotype-specific DMRs. **a** Overlap between the DMRs identified in the NL-NB and CL-CB comparisons. *P*-values derived from Fisher**’**s exact test. **b** Heatmap showing scaled methylation values of the DMRs identified in the NL-NB (left) or CL-CB (right) comparisons. Hierarchical clustering is based on Euclidean distances of the unscaled values and complete linkage. **c** TFBSs enriched in the hypermethylated (purple) or hypomethylated (orange) DMRs common between the NL-NB and CL-CB comparisons. Left panel: analysis performed using HOMER findMotifs, *p*-values from binomial test. Right panel: enrichment of ENCODE defined TFBSs, *p*-values from hypergeometric test against all regions. **d** Frequently hyper- or hypomethylated genes in PCa^7^ that were also hypermethylated (purple) or hypomethylated (orange) in the NL-NB and CL-CB comparisons. **e**, **f** Genome browser plots of the promoter regions of *GSTP1* (**e**) and *CCDC8* (**f**). Grey squares are the bins analysed. Lines and shaded areas represent mean ± SEM of each category (NB = light blue, NL = light red, CB = dark blue, CL = dark red). DMRs are shown on top: hypermethylated = purple, hypomethylated = orange

As suggested by the enriched GO analyses, we found a 28% overlap in all the DMRs identified from the NL-NB and the CL-CB comparisons (3852/13816, Fisher’s exact test *p*-value <10^−300^, Fig. 3a). Hierarchical clustering of all samples based on both sets of DMRs separated them by phenotype (Fig. 3b), reinforcing the presence of a strong phenotypic signature independent of disease state. These shared DMRs were enriched in features characteristic of enhancers (Supplementary Fig. 5A–D) and linked to GO terms related to prostate development, regulation of epithelial stem cells and androgen receptor signalling (Supplementary Fig. 5E, F). Moreover, hypermethylated DMRs were highly enriched for TFBSs of TP63, TP53 and NF1, and hypomethylated DMRs for FOXA1, p65-NFkB and GATA3 (Fig. 3c), all well-known regulators of basal and luminal epithelial cells, respectively. Interestingly, 26 of the 168 genes described as frequently differentially methylated in PCa^7^, showed hyper- or hypomethylated DMRs within 5 kb of their TSSs in both the NL-NB and CL-CB comparisons (Fig. 3d). These included the frequently hypermethylated genes, *GSTP1* and *CCDC8* (Fig. 3e, f).

In summary, these analyses identified a large set of phenotype-specific and disease-independent DMRs, both of which contained many binding sites for TFs with known regulatory roles in the normal prostate.

### CL hypermethylate PRC2 target sites and hypomethylate repetitive elements

**Fig. 4:**
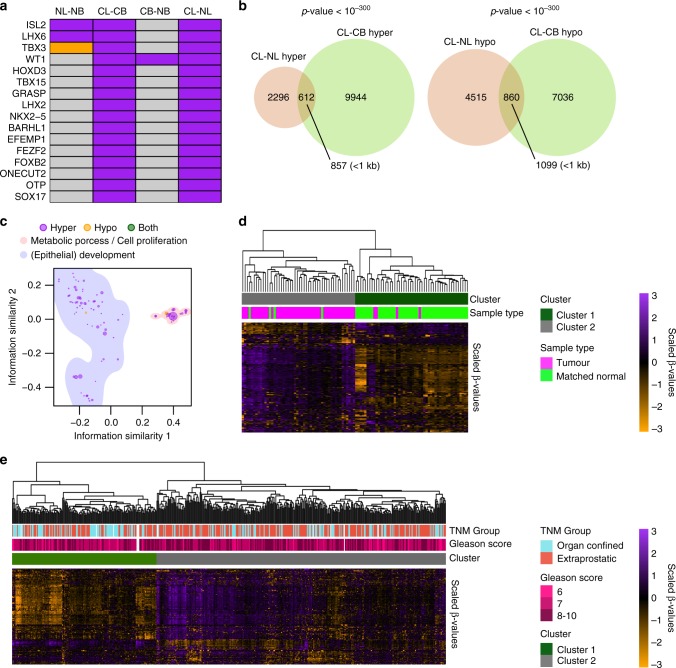
Aberrant methylation in CL. **a** Frequently hyper- or hypomethylated genes in PCa^7^ that are also hypermethylated (purple) or hypomethylated (orange) in the CL-CB and CL-NL comparisons. **b** Overlap between the DMRs identified in the CL-CB and CL-NL comparisons. *P*-values derived from Fisher’s exact test. **c** Clustering of the gene ontologies (biological process) enriched in DMRs common between the CL-CB and CL-NL comparisons based on information similarity. Each circle shows an individual GO term enriched in regions hypermethylated (purple), hypomethylated (orange) or both (green), the size of the circles is proportional to the enrichment *p*-value. The two main clusters of GO terms determined by k-means are highlighted (light blue and pink), and named after the most frequent terms. **d** Heatmap showing scaled methylation values (β-values) of probes overlapping the DMRs common to the CL-CB and CL-NL comparisons in the PCa samples (magenta) and matched normal samples (green) within the TCGA dataset. Hierarchical clustering based on Euclidean distances of the unscaled values and complete linkage. The dark green and grey clusters were generated by cutting the tree at the first bifurcation. **e** Heatmap showing scaled methylation values (β-values) of probes overlapping the DMRs common to the CL-CB and CL-NL comparisons in the PCa samples (matched normal samples not included) of the TCGA dataset. Hierarchical clustering based on Euclidean distance of the unscaled values and complete linkage. The dark green and grey clusters are generated by cutting the tree at the first bifurcation

A second group of genes frequently hypermethylated in PCa were found hypermethylated in both the CL-CB and CL-NL comparisons (Fig. 4a), but not in the NL-NB comparison. These might be expected to reflect a PCa-specific methylation signature. DMRs identified in the CL-CB and CL-NL comparisons showed that many were shared (1472 DMRs, Fisher’s exact test *p*-value < 10^−300^, Fig. 4b) with very few also different between NL and NB cells (106 DMRs). 65% of these CL-specific hypermethylated DMRs were distal to TSSs and were again highly enriched for enhancer features, but significantly depleted in repetitive elements (Supplementary Fig. 6A–E). These regions were associated with GO terms related to metabolic processes, cell proliferation and epithelial development (Fig. 4c) and showed a high enrichment of DNA sequences potentially bound by EZH2 and SUZ12, two main members of the PRC2 complex (Supplementary Fig. 6F). On the other hand, distal hypomethylated DMRs were not enriched for any feature of putative regulatory regions, but significantly overlapped with LINE and LTR elements.

Since the CL subset represents the majority of the cells in untreated PCa samples, we hypothesised that aberrant methylation of these DMRs would be measurable even when whole tissue homogenates are analysed. We therefore interrogated the DNA methylation array dataset for PCa made available by the TCGA consortium, which consists of 50 PCa samples with matched normal counterparts, 452 additional PCa samples without normal counterparts, and 1 metastatic PCa sample.^27^ 255 array probes overlap these 1472 DMRs. Hierarchical clustering of the 50 matched normal and PCa samples showed an almost perfect subdivision based on the malignancy status of the samples (TPR = 0.92, TNR = 0.92, Chi-squared test *p*-value = 2.4 × 10^−16^, Fig. 4d). The same analysis carried out on all 553 samples produced similar results, with one cluster highly enriched in normal samples (Chi-squared test *p*-value = 1.7 × 10^−39^, Supplementary Fig. 6G). This clustering also appeared to divide the PCa samples into two main groups, according to their differences from the normal samples. Exclusive analysis of the cancer samples confirmed this clustering pattern (Fig. 4e) and showed one cluster to be significantly enriched for samples with extraprostatic extensions (pT3 or pT4 in TNM classification, Chi-squared test *p*-value <0.005) in the absence of significant differences in Gleason score (Chi-squared test *p*-value >0.1).

Overall, these results indicate that phenotypic luminal PCa cells possess an aberrant methylation signature characterised by hypermethylation of putative regulatory sequences involved in tissue development, and hypomethylation of LINEs and LTRs repetitive elements. This signature was also able to distinguish cancer samples from normal, and organ-confined from extraprostatic disease.

### Identification of PCa-specific, phenotype-independent DMRs

**Fig. 5:**
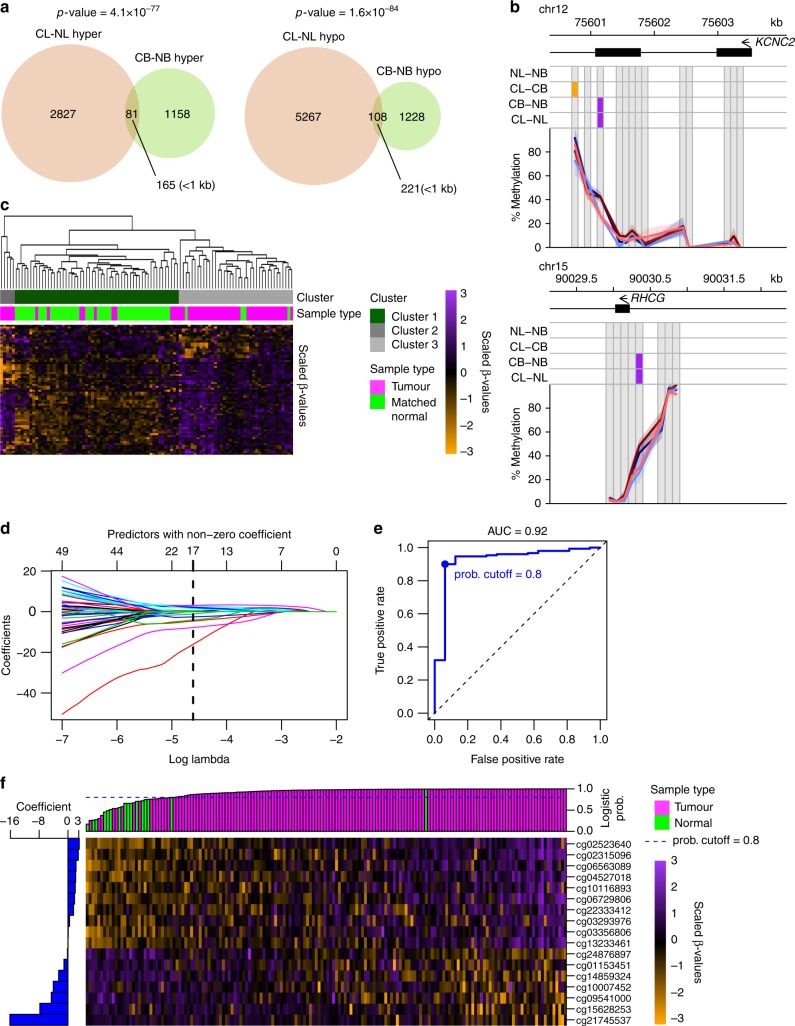
PCa-specific DMRs shared between CB and CL. **a** Overlap between the DMRs identified in the CL-NL and CB-NB comparisons. *P*-values derived from Fisher**’**s exact test. **b** Genome browser views of *KCNC2* promoter (top) and *RHCG* exon 2 (bottom). Grey squares are the bins analysed. Lines and shaded areas represent mean ± SEM of each category (NB = light blue, NL = light red, CB = dark blue, CL = dark red). DMRs are shown on top: hypermethylated = purple, hypomethylated = orange. **c** Heatmap showing scaled methylation values of probes overlapping the DMRs common between CL-CB and CB-NB in the matched normal and cancer samples within the TCGA dataset. Hierarchical clustering based on Euclidean distances of the unscaled values and complete linkage. The dark green and grey clusters were generated by cutting the tree at the first 2 bifurcations. **d** Selection of a 17-probe signature distinguishing normal and PCa samples applying LASSO regression on a logistic model of the training dataset (70% of the TCGA samples). Lines show the changes in coefficients in relation to different lambdas. The vertical dashed line shows the optimal lambda identified using cross-validation. **e** Receiver operating characteristic curve generated by applying the optimal logistic model to the test dataset (30% of the TCGA samples). **f** Heatmap showing scaled methylation values of the 17-probe signature in the test dataset (30% of the TCGA samples). The bar plot on the left side shows the final coefficients for each probe in the model, and the bar plot on top shows the logistic probability generated by for each sample (Green: normal samples, magenta: cancer samples)

Comparisons of the DMRs in the CL-NL and CB-NB pairs showed a small but significant overlap of both hyper- and hypomethylated DMRs in each (189 DMRs in total, Fig. 5a). These common DMRs were able to cluster all samples according to their disease state in a phenotype-independent manner (Supplementary Fig. 7A). Notably, they included DMRs close to many genes previously implicated in prostate cancer (e.g., *NEAT1*, *MTOR*, *RHCG*, *KCNC2*, *WT1*, *HOXC12*, *KMT2B*, Fig. 5b). To determine whether these DMRs would be altered in an independent dataset, we applied the same analysis to the TCGA dataset, where 66 array probes overlapped these 189 DMRs. Hierarchical clustering of the 50 matched normal and PCa samples produced a single cluster containing 46/50 normal samples and 10/50 PCa samples (TPR = 0.8, TNR = 0.92, Chi-squared test *p*-value = 1.8 × 10^−12^, Fig. 5c). Application of the same analysis to all samples in the TCGA database produced similar results: one cluster was highly enriched in normal samples (TPR = 0.87, TNR = 0.74, Chi-squared test *p*-value = 8.3 × 10^−26^, Supplementary Fig. 7B), indicating that at least some of these DMRs are frequently altered in PCa.

To select the probes most strongly associated with disease state (i.e., PCa vs normal), we trained a logistic model using LASSO regression on 70% of the TCGA samples and selected a 17-probe signature (Fig. 5d). We then tested this model on the remaining 30% of the dataset. This resulted in an AUC of 0.92 (TPR = 0.9, TNR = 0.94, Fisher’s exact test *p*-value = 2.82 × 10^−12^ at the selected cut-off of 0.8, Fig. 5e, f, Supplementary Table 4). The 17-probe signature also included sequences proximal to several genes with recognised importance in PCa (e.g., *PLAGL1*/*HYMAI*, *HOXC12*, *KCNC2*), but was completely non-overlapping with other similar signatures recently developed for PCa.^32–36^

## Discussion

PCa is characterised by frequent aberrant DNA methylation of many genomic sites that may contain clinically relevant signatures.^7,37^ The early establishment (presence in pre-neoplastic tissues) and high prevalence of these aberrant patterns is also suggestive of their direct involvement in PCa tumourigenesis. However, the normal prostate epithelium is composed of similar numbers of luminal and basal cells, whereas most treatment-naïve prostate cancers are largely composed of cells with many luminal features. This shift in favour of a transcriptional and epigenomic programme of normal luminal cells might mask or complicate the identification of cancer-specific features in prostate cancer when bulk analyses are performed on this type of tumour.

We now report a detailed comparison of genome-wide methylation profiles obtained separately from epithelial cells with luminal and basal phenotypes, isolated with a high purity from patient-matched normal and cancer biopsy samples. From comparative analyses of these profiles, we found a major proportion of the methylation differences between normal basal and luminal cells were conserved in their malignant counterparts. These affected many promoters frequently described as aberrantly methylated in bulk PCa compared to normal tissues, consistent with the increased representation of cells with a luminal phenotype in PCa, in which a higher proportion of cells carrying a methylation signature of normal luminal cells might then be expected.

However, our study made it possible to identify, for the first time, regions specifically altered in the luminal fraction of PCa. The hypermethylated DMRs in this group were associated to genes involved in metabolic processes, cell proliferation and epithelial development, all functions clearly deregulated in prostate cancer, therefore potentally containing major cancer driver events. Furthermore, hypomethylated DMRs were highly enriched in repetitive elements, a feature also previously reported in many cancer types, where they have been thought to contribute to genomic instability and aberrant gene expression.^38–40^

Importantly, this set of DMRs was able to discriminate not only normal and PCa samples in the TCGA dataset, but also PCa samples with or without extraprostatic extensions, the former being indicative of highly aggressive, invasive cancers. Since this distinction was not evident from the Gleason grades of these tumours, the epigenetic data may reflect an acquisition of specific aberrant epigenomic changes that herald disease progression.^7,41–43^ Genomic regions consistently altered in both tumour phenotypes in the PCa samples analysed also have potential clinical importance. Indeed, the new logistic model constructed from these regions makes use of only 17 probes to distinguish normal and PCa samples with similar specificity and sensitivity to previously developed, non-overlapping models,^35,36^ and may be useful in the context of the low mutagenic burdens seen in most hormone-naïve prostate cancers.

The results reported here show that many DNA methylation changes commonly associated with PCa cells are explained by a predominant luminal phenotype of the treatment-naïve PCa population, and are not cancer-specific nor are likely to contain driver events. Importantly however, we were able to identify two separate classes of PCa-specific DNA methylation changes: those specific to cancer luminal cells that can distinguish both normal from cancer samples and organ-confined cancers from those with extraprostatic extensions; those common to basal and luminal cancer cells that are able to distinguish PCa efficiently from normal samples. These two novel sets of cancer-specific changes clearly demonstrate the potential of profiling normal and cancer cell subpopulations in identifying signatures that may contain previously unrecognised driver events in the development and progression of PCa.

## Electronic supplementary material

Supplementary Figures and Legends

Supplementary Table 1

Supplementary Table 2

Supplementary Table 3

Supplementary Table 4

## Availability of data and material

The methylation and coverage calls for all RRBS libraries generated are available from GEO [GSE107596]. For patients’ privacy reasons, raw data (FASTQ and BAM files) for the RRBS libraries are not publicly available, but can be available from the corresponding author on request.
