# Supplementary material for: Phenotype-independent DNA methylation changes in prostate cancer
Source: Br J Cancer. 2018 Oct 15;119(9):1133–43. doi: 10.1038/s41416-018-0236-1 (PMC6219500; doi:10.1038/s41416-018-0236-1)
Supplement: Supplementary file 1 — Supplementary Figures and Legends [file 41416_2018_236_MOESM1_ESM.pdf]

**Supplementary Files:**

## Supplementary Figure 1

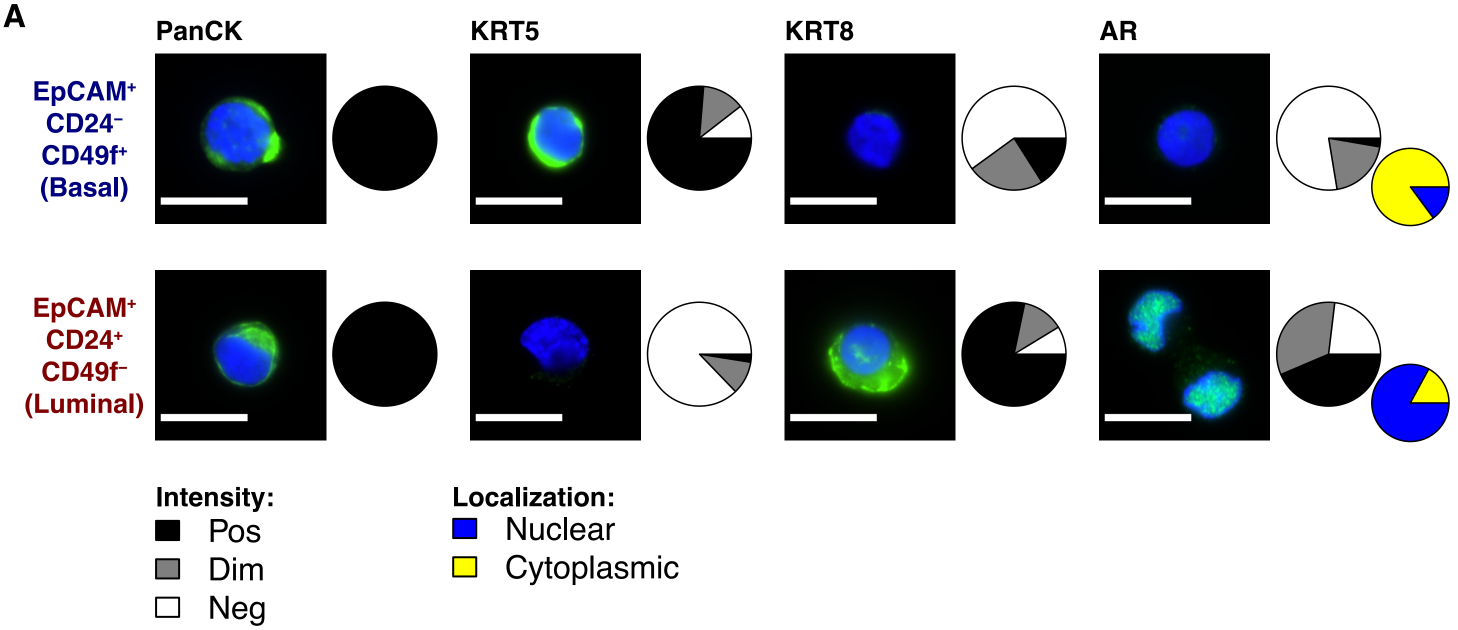


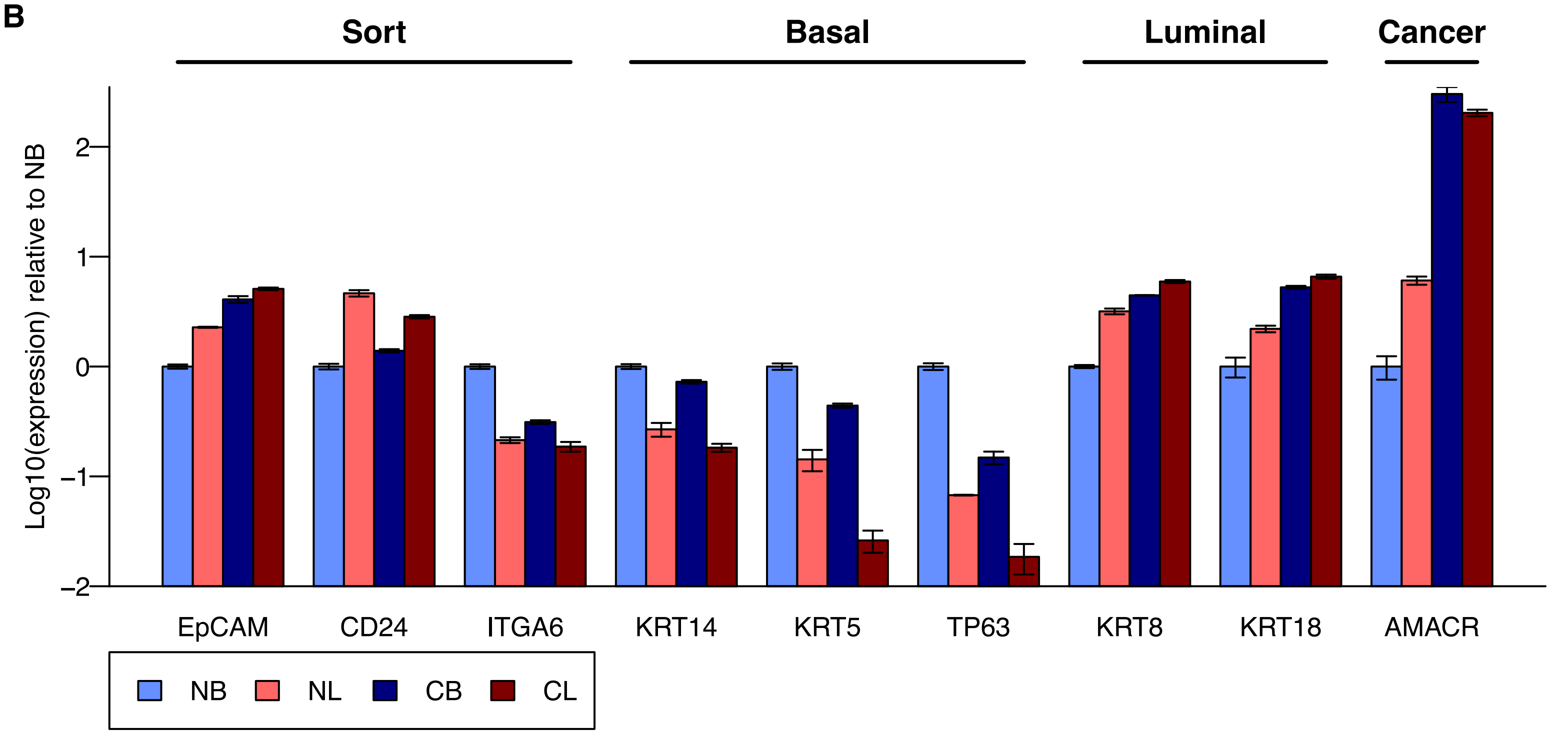


C

| Donor | Age | Gleason | PSA | Cancer Positive Biopsy Cores | Palpable Tumour |
|---|---|---|---|---|---|
| H329 | 53 | 3+4 | 51 | Right 0/5 - Left 3/5 | Left base |
| H309 | NA | 3+4 | 9.1 | Right 0/5 - Left 2/5 | Left apex |
| H323 | 64 | 3+4 | 20.4 | Right 6/7 - Left 7/8 | Left side and right apex |
| H324 | 62 | 3+4 | 3.9 | Right 4/5 - Left 0/5 | Right side |

**Supplementary Fig. 1: Validation of the FACS sorting strategy for basal and luminal cells.** (**A**) Immunofluorescence for pan-cytokeratin (PanCK), cytokeratin 5 (KRT5), cytokeratin 8 (KRT8) and androgen receptor (AR) on cytospin preparations of sorted EpCAM+CD49f+CD24- and EpCAM+CD49f-CD24+ cells. The pie charts show the proportion of negative (white) dim (grey) and bright (black) cells for each marker. At least 100 cells per population per marker were counted. For AR, the fraction of dim or positive cells with cytoplasmic (yellow) or nuclear (blue) localization is also shown. One representative image is shown for each marker in the green channel, and DAPI counterstain in blue. Scale bars = 10 μm. (**B**) qRT-PCR analysis performed on FACS sorted EpCAM+CD49f+CD24- and EpCAM+CD49f-CD24+ cells from one matched tumor directed and contralateral biopsy. Bar show mean ±SEM of the log10(ΔΔCt values) using GAPDH as housekeeping gene as NB population as a reference sample. (**C**) Table of the pre-operation clinical features of the donors for the samples used for the DNA methylation analysis (PSA measured in ng/ml)

## Supplementary Figure 2

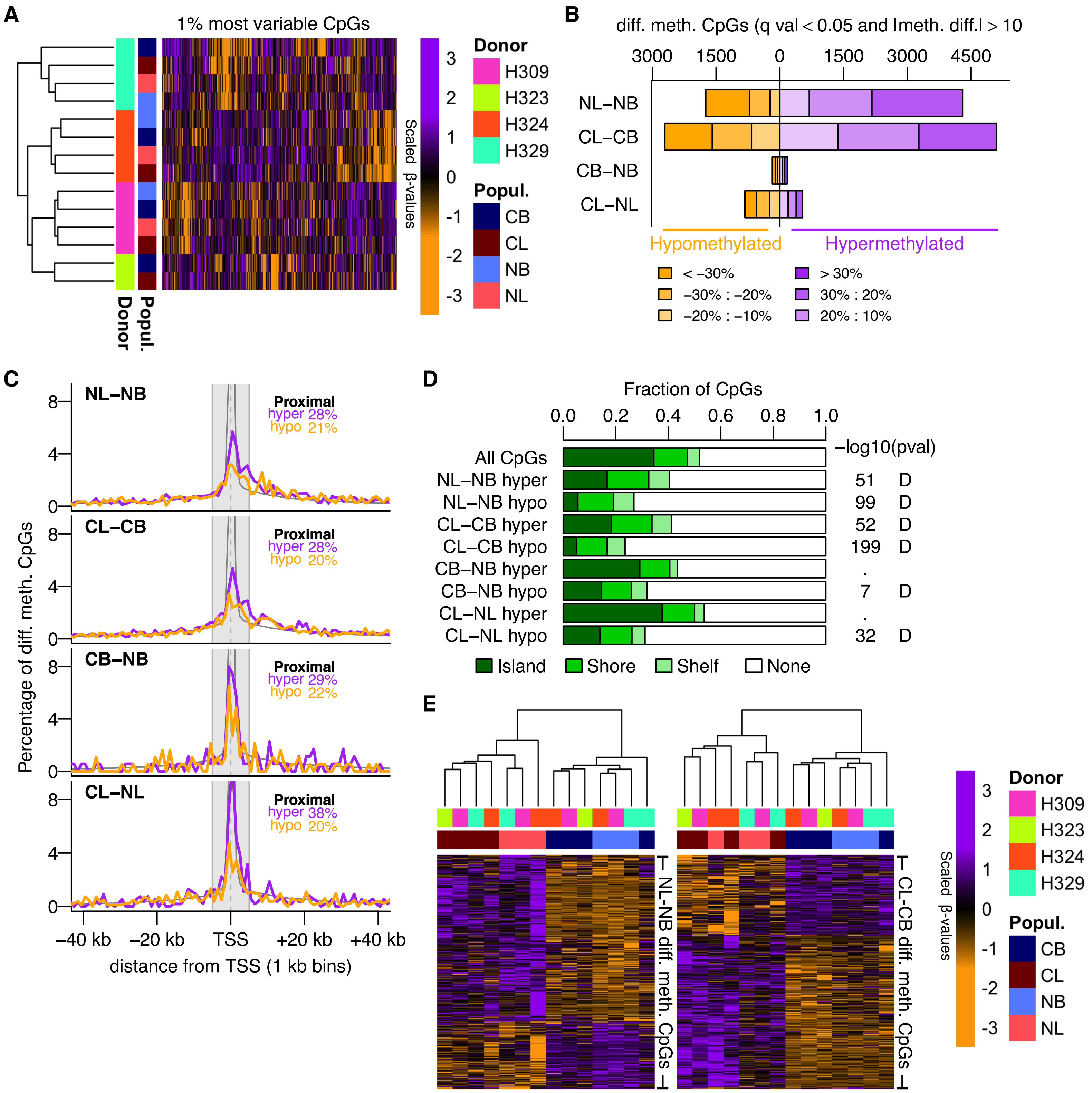

**Supplementary Fig. 2: Identification of differentially methylated CpGs between prostate cancer cell populations.** (**A**) Heatmap showing scaled methylation values of the top 1% most variable CpGs in the samples analysed. Hierarchical clustering is based on Euclidean distance of the unscaled values and complete linkage. (**B**) Number of differentially methylated CpGs found in each comparison. (**C**) Distribution of distances of differentially methylated CpGs to the closest TSS. Grey box indicates ±5 kb from a TSS. Purple lines: hypermethylated, orange lines: hypomethylated , gray line: all CpGs. (**D**) Overlap of differentially methylated CpGs with CpG islands, shores (2 kb flanking islands) or shelves (2 kb flanking shores). P-values from hypergeometric test against all CpGs analysed. E = enriched, D = depleted. (**E**) Heatmap showing scaled methylation values of the differentially methylated CpGs identified in the NL-NB (left) or CL-CB (right) comparisons. Hierarchical clustering is based on Euclidean distances of the unscaled values and complete linkage.

## Supplementary Figure 3

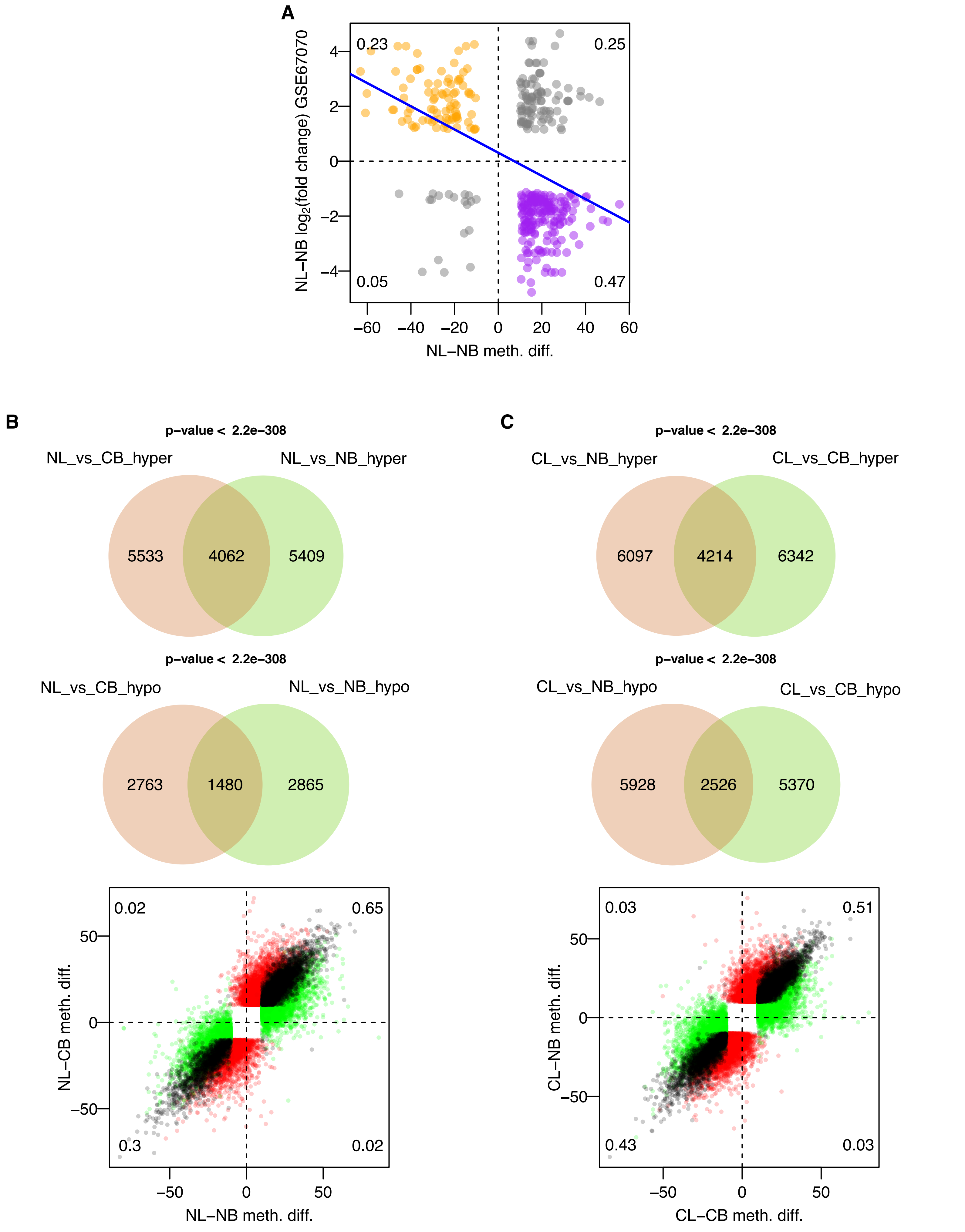

**Supplementary Fig. 3: Proximal DMRs are associated with differential expression.** (**A**) Dot-plot showing for each differentially expressed gene associated with a proximal (<5 kb from TSS) DMR in NL-NB differential methylation (x axis) and differential expression (y axis) in similarly selected cell populations. Each dot represents one gene/DMR association; purple dots: hypermethylated and downregulated genes; orange dots: hypomethylated and upregulated genes; blue line: least squares linear fit. (**B**) Top: Venn diagrams showing the overlap of the DMRs obtained in the NL-NB and NL-CB comparisons (p-values from Fisher's exact test). Bottom: dot-plot showing the methylation difference of the DMRs identified in the NL-NB (green), NL-CB (red) comparisons, or both (Black). (**C**) Top: Venn diagrams showing the overlap of the DMRs obtained in the CL-CB and CL-NB comparisons. Bottom: dot-plot showing the methylation difference of the DMRs identified in the CL-CB (green), CL-NB (red) comparisons, or both (Black).

## Supplementary Figure 4

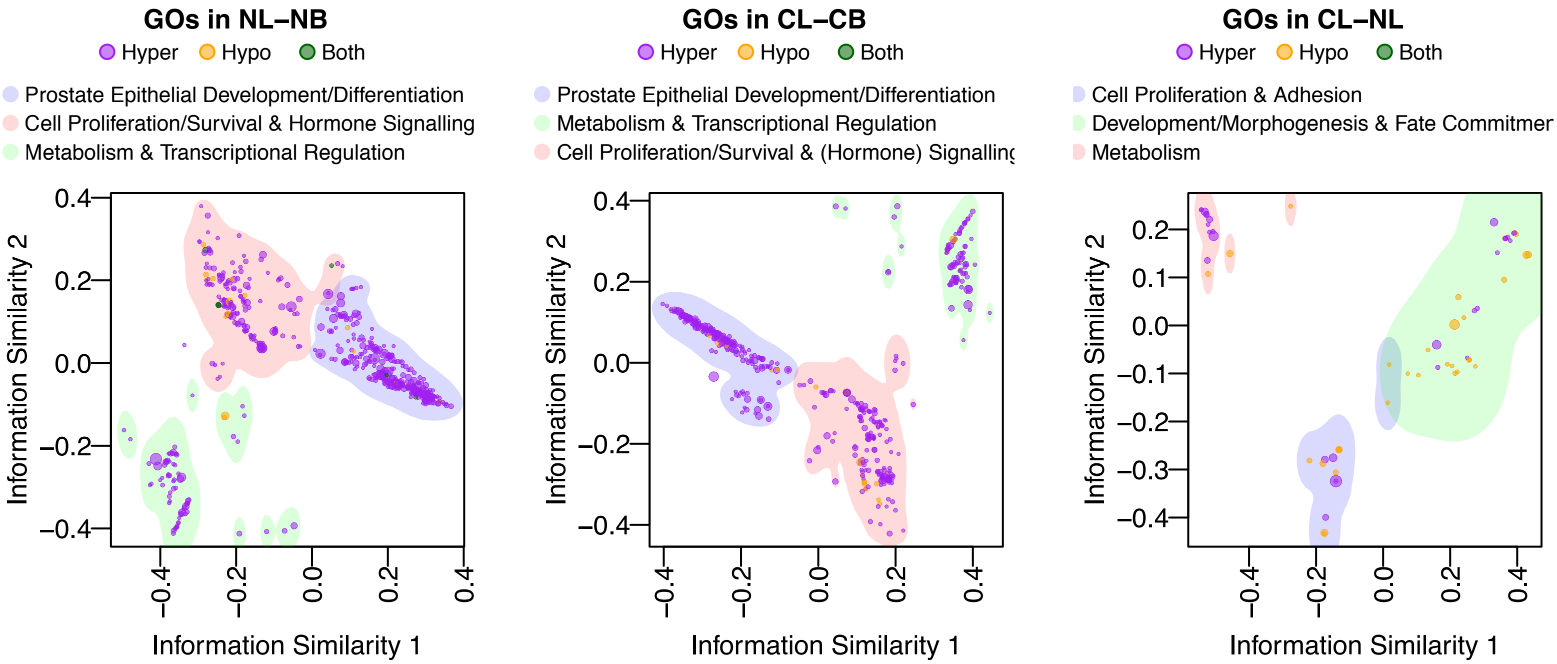

**Supplementary Fig. 4: Gene ontology enrichment analysis.** Clustering of the gene ontologies (biological process) enriched in DMRs identified in the NL-NB (left) CL-CB (middle) and CL-NL (right) comparisons based on information similarity. Each circle shows an individual GO term, the size of the circles is proportional to the enrichment p-value. The 3 main clusters of GO terms determined by k-means are highlighted.

**Supplementary Figure 5**

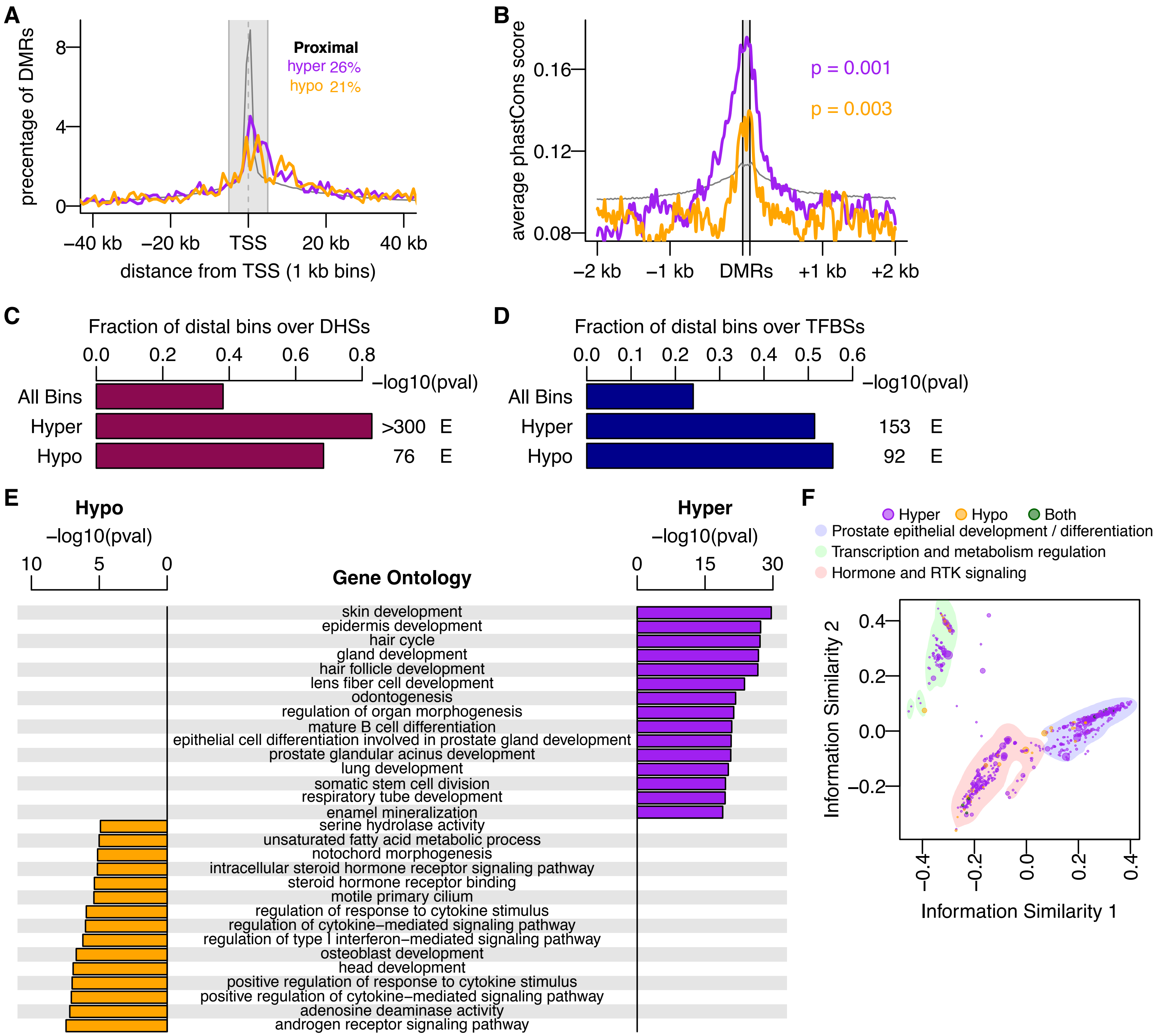

**Supplementary Fig. 5: Phenotype-specific distal DMRs are highly enriched in enhancer features.** (**A**) Distribution of distances of DMRs common between the NL-NB and CL-CB comparisons to the closest TSS. Grey box indicates ±5 kb from TSS. Purple line: hypermethylated DMRs, orange line: hypomethylated DMRs, gray line: all regions. (**B**) Average plots of evolutionary conservation scores of the distal DMRs common between the NL-NB and CL-CB comparisons. Purple line: hypermethylated DMRs, orange line: hypomethylated DMRs, gray line: all regions. P-values from bootstrapping analysis. (**C**) Proportion of distal DMRs common between the NL-NB and CL-CB comparisons that overlapped with DHSs (identified by ENCODE). P-values from hypergeometric test against all regions. E = enriched, D = depleted. (**D**) Overlap of distal DMRs common between the NL-NB and CL-CB comparisons with ChIP-seq-derived TFBSs (identified by ENCODE). P-values from hypergeometric test against all regions. E = enriched, D = depleted. (**E**) Top 15 gene ontologies enriched in hypermethylated and hypomethylated DMRs common between NL-NB and CL-CB. P-values from hypergeometric test (FDR<0.05 and at least 3 genes in the set). (**F**) Clustering of the gene ontologies (biological process) enriched in DMRs common between the NL-NB and CL-CB comparisons based on information similarity. Each circle shows an individual GO term, the size of the circles is proportional to the enrichment p-value. The 3 main clusters of GO terms determined by k-means are highlighted.

## Supplementary Figure 6

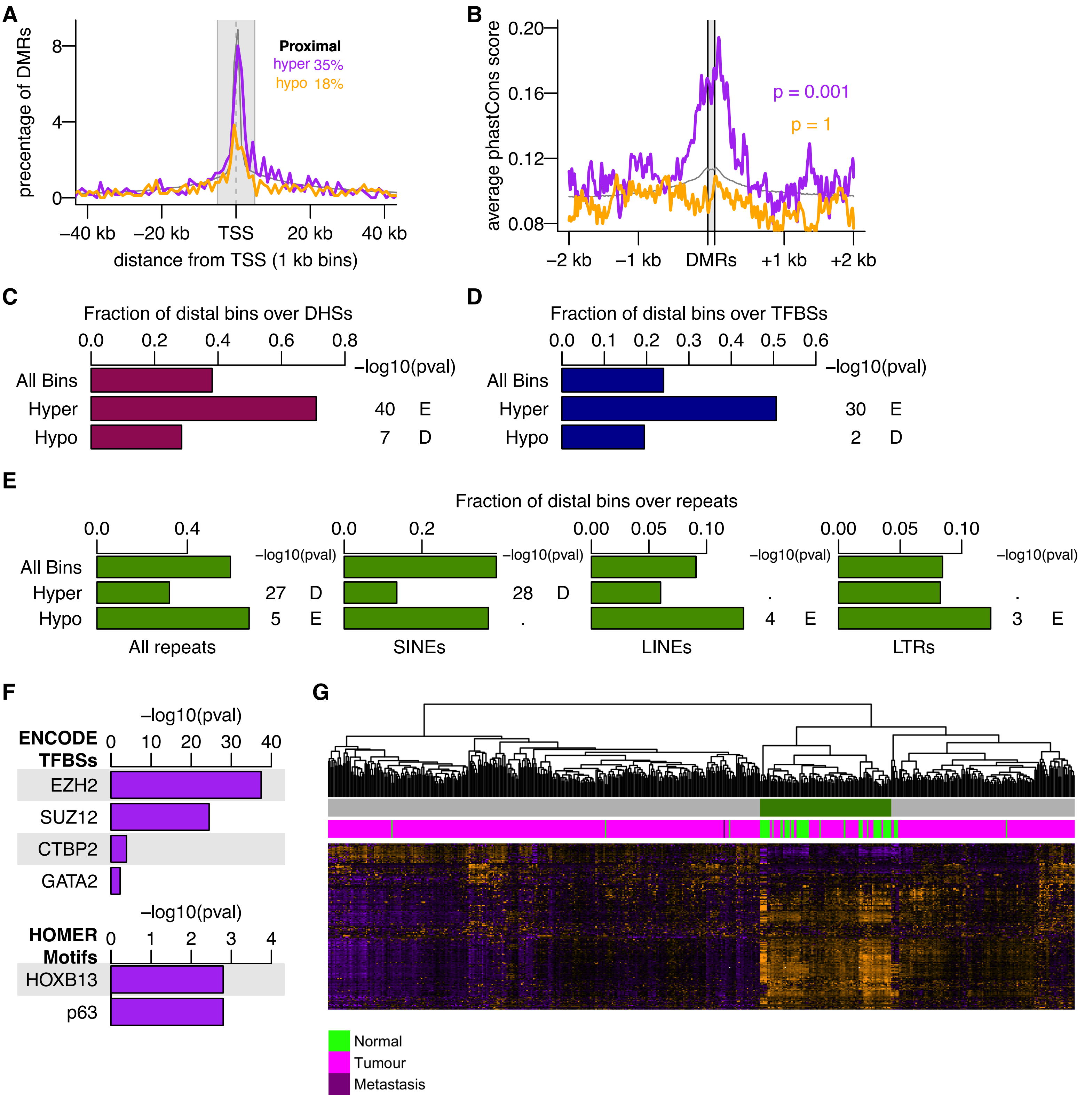

**Supplementary Fig. 6: Aberrant methylation in luminal cells from PCa samples.** (**A**) Distribution of distances of the DMRs common between the CL-CB and CL-NL comparisons to the closest TSS. Grey box indicates ±5 kb from TSS. Purple line: hypermethylated DMRs, orange lines hypomethylated DMRs, gray line: all regions. (**B**) Average plots of evolutionary conservation scores of the distal DMRs common between the CL-CB and CL-NL comparisons. Purple line: hypermethylated DMRs, orange lines hypomethylated DMRs, gray line: all regions. P-values from bootstrapping analysis. (**C**) Proportion of distal DMRs common between the CL-CB and CL-NL comparisons that overlapped with DHSs (identified by ENCODE). P-values from hypergeometric test against all regions. E = enriched, D = depleted. (**D**) Overlap of distal DMRs common between the CL-CB and CL-NL comparisons with ChIP-seq-derived TFBSs (identified by ENCODE). P-values from hypergeometric test against all regions. E = enriched, D = depleted. (**E**) Overlap of each set of distal DMRs common between the CL-CB and CL-NL comparisons with repetitive elements (UCSC repeatMask), SINEs, LINEs and LTRs. P-values from hypergeometric test against all regions. E = enriched, D = depleted. (**F**) TFBSs enriched in the DMRs common between the CL-CB and CL-NL comparisons. Top panel: enrichment of ENCODE defined TFBSs, p-values from hypergeometric tests against all regions. Bottom panel: analysis performed using HOMER findMotifs, p-values from binomial tests. (**G**) Heatmap showing scaled methylation values of probes in the TCGA dataset (all samples) overlapping the DMRs common between the CL-CB and CL-NL comparisons. Hierarchical clustering based on Euclidean distance of the unscaled values and complete linkage. The dark green and gray clusters are generated by cutting the tree at the first 2 bifurcations.

## Supplementary Figure 7

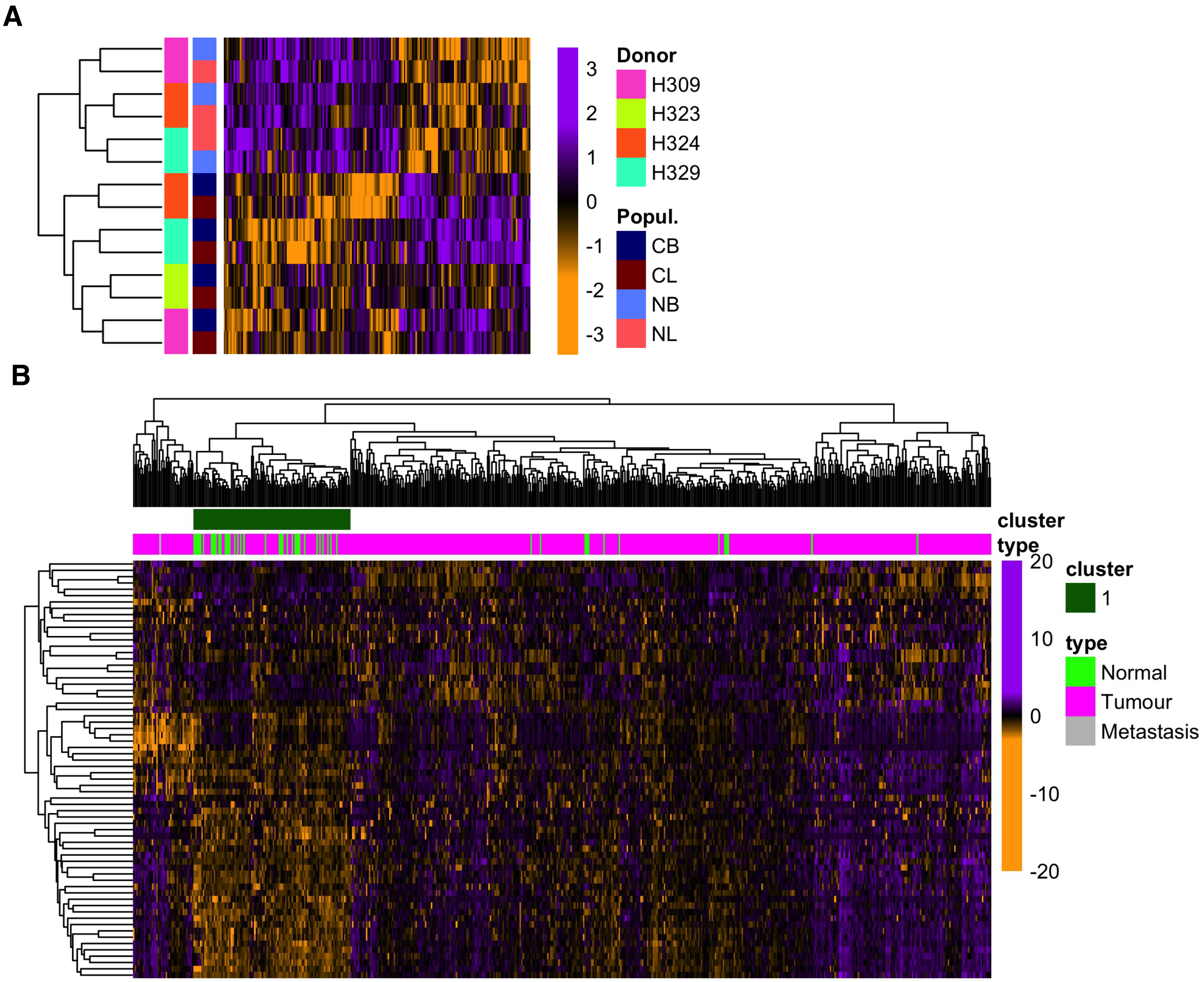

**Supplementary Fig. 7: PCa-specific DMRs shared by both basal and luminal subsets.** (**A**) Heatmap showing scaled methylation values of the DMRs common between the CL-CB and CB-NB comparisons. Hierarchical clustering is based on Euclidean distance of the unscaled values and complete linkage. (**B**) Heatmap showing scaled methylation values of probes in the TCGA dataset (all samples) overlapping the DMRs common between the CL-CB and CB-NB comparisons. Hierarchical clustering based on Euclidean distance of the unscaled values and complete linkage. The dark green and gray clusters are generated by cutting the tree at the first 3 bifurcations.

**Supplementary Table 1:** Quality metrics for all RRBS libraries generated.

**Supplementary Table 2:** Fractional methylation, coverage, differential methylation and adjusted p-values for all DMRs.

**Supplementary Table 3**: Fractional methylation, coverage, differential methylation and adjusted p-values for all differentially methylated CpGs.

**Supplementary Table 4**: 17-CpG methylation signature.
