# Supplementary material for: Phenotype-independent DNA methylation changes in prostate cancer
Source: Br J Cancer. 2018 Oct 15;119(9):1133–43. doi: 10.1038/s41416-018-0236-1 (PMC6219500; doi:10.1038/s41416-018-0236-1)
Supplement: Supplementary file 5 — Supplementary Table 4 [file 41416_2018_236_MOESM5_ESM.pdf]

chr	start	end	Probe ID	Closest TSS	Distance to TSS	CpG island / shore / shelf
chr1	20569455	20569456	cg22333412	NM_001039500	-47956	Island
chr1	20988602	20988603	cg24876897	NM_005216	-565	Shore
chr10	129534612	129534613	cg02523640	NM_207426	-925	Island
chr11	121233742	121233743	cg21745537	NM_001024956	70166	Island
chr11	121233756	121233757	cg15628253	NM_001024956	70180	Island
chr12	54350208	54350209	cg13233461	NM_173860	1495	Shore
chr12	54350293	54350294	cg06729806	NM_173860	1580	Shore
chr12	75601156	75601157	cg06563089	NM_001260497	2372	Island
chr13	110522019	110522020	cg02315096	NM_003749	-83105	Island
chr14	105714273	105714274	cg01153451	NM_145685	-51	Shore
chr15	26874362	26874363	cg14859324	NM_001191321	-37	Island
chr17	9019123	9019124	cg10116893	NR_110828	63312	Island
chr20	42143488	42143489	cg09541000	NM_015478	413	Island
chr3	126261297	126261298	cg04527018	NM_152533	16461	Island
chr3	152554194	152554195	cg03293976	NM_002563	1459	Shore
chr6	144329330	144329331	cg10007452	NM_001080952	211	Island
chrX	120181819	120181820	cg03356806	NM_012084	358	Island
